# When the decision to die interferes with the duty to heal

**DOI:** 10.1186/s13690-025-01615-2

**Published:** 2025-05-07

**Authors:** Maya Morcos, Amir-Ali Golrokhian-Sani

**Affiliations:** Faculty of Medicine, University of Ottawa, Ottawa, ON Canada

## Abstract

**Background:**

This commentary was inspired by an encounter M. M. experienced while shadowing a physician in 2024. The physician referred an otherwise healthy patient between 64 and 74 years old for a routine colonoscopy due to relevant risk factors. However, instead of the anticipated report, they received a letter from the specialist stating their refusal to complete the procedure. The reason cited for refusal: medical assistance in dying (MAiD). In the meeting with the specialist, the patient mentioned that they were considering pursuing MAiD for depression in 2026 - a choice that, notably, would not be available for solely mental health conditions until March 17, 2027.

**Results/Conclusion:**

Here, we consider multiple angles centred around how we should treat MAiD, particularly when it intersects with decisions related to life expectancy. Policy reform is necessary to address this potential form of discrimination across all subspecialties in medicine, advocating instead for collaborative, case-by-case decision-making between physicians and patients to discuss their goals of care and risks. To this end, we propose a four-pronged approach, including guidelines, medical ethics training, patient-targeted education, and further research.

**Table:** 

**Text box 1. Contributions to the literature**
• This paper highlights how provider biases on MAiD may influence access to care.
• No research has examined whether patients pursuing MAiD experience inequitable healthcare access in the Canadian setting. This paper aims to contribute to this discussion through a real-world example.
• This paper argues that a plan to pursue MAiD should not be considered a contraindication to routine medical procedures for patients.
• This paper advocates for expanded medical guidelines ensuring that MAiD considerations do not limit patient access to care.

This essay was inspired by an encounter M. M. experienced while shadowing a physician in 2024. The physician referred an otherwise healthy patient between 64 and 74 years old for a routine colonoscopy due to relevant risk factors. However, instead of the anticipated report, they received a letter from the specialist stating their refusal to complete the procedure. The reason cited for refusal: medical assistance in dying (MAiD). In the meeting with the specialist, the patient mentioned that they were considering pursuing MAiD for depression in 2026 - a choice that, notably, would not be available in Canada for solely mental health conditions until March 17, 2027 [1]. Herein, we see multiple angles for consideration centred around how we should treat MAiD, particularly when it intersects with decisions related to life expectancy. What made this case perplexing was that the patient was capable of completing the colonoscopy preparation, and their medical history did not indicate any urgent or life-threatening conditions that would render the procedure inadvisable. Thus, the refusal was based not on medical necessity but on the assumption that the patient’s consideration of MAiD should influence their present care.

In 2021, MAiD laws in Canada were revised by Bill C-7 to include eligibility for patients suffering solely from mental illness, starting March 17, 2023. However, Bill C-39 delayed the implementation of this law until March 17, 2024, with the hope of implementing proper safeguards and training [2]. This legislation was further delayed until March 17, 2027, following consultations stating that the healthcare system was not yet prepared for such an undertaking. Despite this law not coming into effect as of yet, many patients have started considering MAiD for solely mental illness as a future option [2]. Thus, the healthcare system needs to adapt quickly to ensure appropriate and equitable treatment for such groups.

This case raises several questions concerning the collateral effects of MAiD. Firstly, one must consider whether patients who are considering or pursuing MAiD are accessing quality healthcare. This is a core consideration of this paper, as no research exists on inequitable treatment for MAiD patients in Canada. It is, naturally, a challenging qualitative study to undertake, given that patients can only be interviewed before the procedure. This case is simply one example of inequitable treatment, with the lack of large-scale data on the subject leaving the magnitude of this question unanswered. From the perspective of the healthcare worker, there may be an emotional toll tied to treating someone who wants to end their life. If the healthcare team expects the patient to pursue MAiD, this may make curative treatment feel less worthwhile or fulfilling. There is a possibility that they may be inclined to neglect more effort-intensive interventions, providing fewer options to the patient, leading to suboptimal care and increased system strain.

The second important consideration regards whether a patient, upon further reflection or changes in health, decides against pursuing MAiD.The Canadian 2022 annual report on MAiD found that 1.9% of written requests for MAiD were withdrawn, with the majority (75.9%) being due to the requester changing their mind [3]. Moreover, 15.6% of these withdrawals occurred immediately before the procedure.^3^ Furthermore, in a cohort of 48 euthanasia-approved patients in Belgium for solely psychological suffering, eight postponed or cancelled their procedure [4]. Ultimately, changing one’s mind about MAiD can, and does, occur at any point. If a healthcare practitioner were to treat MAiD as if it were inevitable, the patient may feel more pressured to follow through despite their hesitation. A major end-of-life concern for individuals accessing services like MAiD is being a burden on family, friends, or caregivers [5]. Patients are, therefore, at risk of feeling like changing their mind is an inconvenience if the physician treats it like a certainty instead of a possibility.

Additionally, one must consider whether a treatable pathology is compromising a patient’s quality of life, pushing them to pursue MAiD. For instance, a 2022 systematic review and meta-analysis found a significant association between colorectal cancer and depression (pooled HR 1.78; 95% CI 1.23 to 2.57) [6]. Although some risks for depression (e.g., cancer treatment) do not apply to the patient who inspired this essay, others do (e.g., clinical and sociodemographic characteristics) [6]. It is, therefore, possible that the patient’s depression was somewhat influenced by a pathological source, which could have been caught and treated through a colonoscopy. With the excessive wait times for various health services in Canada, cases like this may slip through the cracks [7].

Overall, these considerations culminate in one question: Should the consideration or pursuit of MAiD be a contraindication to screening procedures? Building off the previous three questions, MAiD should not be considered a contraindication to screening procedures. Unlike terminal illness, MAiD can be stopped voluntarily and may have an addressable root cause. Patients can still be treated for various diseases while pursuing MAiD, as the two are not mutually exclusive. The implementation of this legislation has already been delayed twice, from 2023 to 2024 and now to 2027. It is, therefore, unwise to base clinical decisions on a law that is subject to ongoing debates and an uncertain future direction.

Being refused a potentially life-saving procedure based solely on a patient’s future consideration of death reflects a bias that compromises the quality of patient care across Canada, which may reinforce systemic inequities and societal stigma concerning mental health. The contemplation of MAiD is not synonymous with a terminal illness or the certainty of death. Unfortunately, this case is not a standalone example of limited patient care as a result of treatment preferences. For instance, a study showed that nurses are significantly less likely to call a physician or a rapid response when their patient undergoes certain changes (e.g., tachycardia, mental status changes) if they are labeled as “do not resuscitate” [8]. This case is a new iteration of an old problem, highlighting an emerging form of a systemic trend in care-limiting bias that has not yet been researched in the clinical context. We urge the Canadian medical system to adapt to this under-examined issue, which may become more prevalent in the coming years. Policy reform is necessary to address this potential form of discrimination across all subspecialties in medicine, advocating instead for collaborative, case-by-case decision-making between physicians and patients to discuss their goals of care and risks. To this end, we propose a four-pronged approach:

## Guidelines

MAiD requests can be stopped at any time, so they should not be treated as a determinant of life expectancy. Future decisions concerning end-of-life care should be kept distinct from medical care and vice versa. Guidelines should explicitly except the pursuit of MAiD as an absolute contraindication. This would standardize the guidelines regarding the interplay between MAiD and medical care, reducing diagnostic delays and promoting consistent health outcomes.

## Medical ethics training

Comprehensive training in medical ethics should be provided to healthcare workers to accompany the changes in the legislature concerning MAiD. Providers should be informed that MAiD withdrawal is a very real possibility and be urged to discuss their patients’ individual goals of care. This would help to reduce provider uncertainty, minimize inconsistent decision-making, and dismantle prejudices that could impact clinical choices.

## Patient-Targeted education

Communication protocols need to be refined and robust, with patient-targeted informational resources that would help teach them about their rights to access care, encourage them to exercise their right to autonomy, and guide the appeal process if they are unjustly denied medical access. By helping patients understand their options and learn to navigate the system, the potential for worsened outcomes is minimized.

## Further research

Studies should be conducted to assess how MAiD influences a patient’s access to healthcare. Individuals and their support groups should be interviewed about the process and their medical interactions once labeled as someone pursuing MAiD. Physicians and healthcare providers should be interviewed concerning their views on the emotional toll of treating a patient pursuing MAiD. This would help inform future policy developments, ultimately leading to more effective and humane healthcare.

The takeaway point from this case is the recognition that an individual can change their mind about MAiD at any time. Therefore, until the process is complete, there is no certainty that they will be dying any sooner than would be predicted naturally. To refuse a procedure based on a future choice is to project a dangerous assumption about the patient’s life trajectory. Physicians are bound by a duty to act in their patients’ best interests, and we urge them not to let uncertain assumptions or biases sway their clinical decision-making. After all, it is the duty of a doctor to defy death, not to surrender to it prematurely.

## Data Availability

No datasets were generated or analysed during the current study.
